# The Ecdysone receptor constrains wingless expression to pattern cell cycle across the *Drosophila* wing margin in a cyclin B-dependent manner

**DOI:** 10.1186/1471-213X-13-28

**Published:** 2013-07-13

**Authors:** Naomi C Mitchell, Jane I Lin, Olga Zaytseva, Nicola Cranna, Amanda Lee, Leonie M Quinn

**Affiliations:** 1 Department of Anatomy and Cell Biology, University of Melbourne, Parkville 3010, Melbourne, Australia

**Keywords:** Ecdysone, Cyclin B, Drosophila, Cell cycle, Wingless

## Abstract

**Background:**

Ecdysone triggers transcriptional changes via the ecdysone receptor (EcR) to coordinate developmental programs of apoptosis, cell cycle and differentiation. Data suggests EcR affects cell cycle gene expression indirectly and here we identify Wingless as an intermediary factor linking EcR to cell cycle.

**Results:**

We demonstrate EcR patterns cell cycle across the presumptive *Drosophila* wing margin by constraining *wg* transcription to modulate CycB expression, but not the previously identified Wg-targets dMyc or Stg. Furthermore co-knockdown of Wg restores CycB patterning in EcR knockdown clones. Wg is not a direct target of EcR, rather we demonstrate that repression of Wg by EcR is likely mediated by direct interaction between the EcR-responsive zinc finger transcription factor Crol and the *wg* promoter.

**Conclusions:**

Thus we elucidate a critical mechanism potentially connecting ecdysone with patterning signals to ensure correct timing of cell cycle exit and differentiation during margin wing development.

## Background

Metamorphosis of *Drosophila* involves proliferation, differentiation and death of larval tissues in order to form the adult fly. The major developmental hormone in *Drosophila*, the steroid hormone 20-hydroxyecdysone (ecdysone) is secreted from the prothoracic gland (PG) in pulses that precede critical morphological changes during development [1-4]. Ecdysone pulses are required for all aspects of developmental timing and morphogenesis, starting with the formation of the body plan during late embryogenesis required to develop to the first instar larval stage and for the cuticle moulting at the end of the first and second instars. A large titre of ecdysone is released at the end of the third larval instar in preparation for pupation, which marks the beginning of adult tissue metamorphosis [1,2]. Metamorphosis is orchestrated by the cascade of gene transcription triggered by ecdysone, which activates the ecdysone receptor (EcR), a member of the nuclear receptor family [1,2].

**Figure 1:**
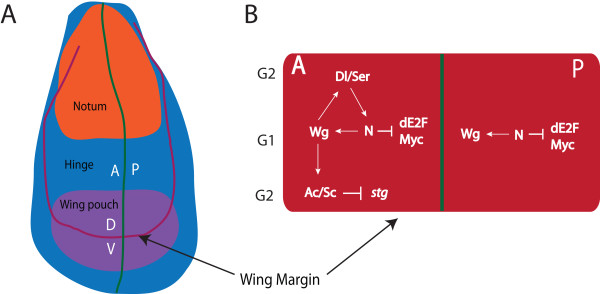
**Patterning cell cycle across the wing imaginal disc. (A)** - Schematic of the 3rd instar wing disc. The red and blue region develops to form the notum and hinge while the purple region forms the wing blade. The green line marks the anterior-posterior (A/P) boundary while the red line defines the dorsal-ventral (D/V) boundary. **(B)** - Across the margin Notch (N) expression triggers the activation of Wingless (Wg). In the margin Wg induces G1 delay via repression of dE2F1 activity in a narrow domain within the anterior compartment and also in a broader region of the posterior compartment. In the anterior compartment, Wg also induces G2 delay in the cells flanking the G1 band by down regulating expression of *stg*.

The *Drosophila* larval wing imaginal disc has long served as an excellent system to elucidate connections between the activity of developmental signals and patterning of cell cycle gene expression, but potential mechanism(s) modulating these events via ecdysone/EcR remain a mystery. The wing disc is comprised of an epithelial sheet, which can be divided into distinct domains based on cell fate in the adult wing; the notum, hinge and pouch (Figure 1A). With the release of the ecdysone hormone at the end of the third instar, proliferation of the wing imaginal disc slows and differentiation of the adult sensory neurons begins along the presumptive wing margin [5,6]. Cell division is tightly coupled with differentiation in the cells comprising the wing margin, which undergo a cell cycle delay in order to pattern proneural gene expression in the clusters of sensory neuron precursor (SOP) cells required for differentiation and development of bristles [7,8]. However, a subset of margin cells must remain competent to re-enter the cycle as bristle precursors do not complete their final cell divisions until 24 hours After Puparium Formation (APF), by which time all epithelial cells of the wing have exited the cell cycle and most cells have arrested in G1 [5,6]. Thus for proper timing of wing margin development, cells spanning the dorsal-ventral (D/V) boundary must first undergo a coordinated cell cycle delay, but must also be competent to re-enter the cell cycle to complete bristle cell divisions during early pupal stages.

Interactions between the Wingless (Wg) secreted morphogen and the Notch (N) receptor pathway have been implicated in this cell cycle patterning across the presumptive wing margin [9-12]. Although all cells at the D/V boundary are cell cycle delayed during the late third instar, patterning between the anterior and posterior compartments differs; posterior cells both within and immediately flanking the D/V boundary are delayed in G1, while the G1 band across the anterior of the boundary is flanked by G2 delayed cells (Figure 1B) [9-11,13,14]. During the transition from second to third instar, the D/V boundary is established through the activity of Notch signaling, via the Notch ligands Serrate and Delta, which lead to activation of *wg* expression [15-17]. Through an auto-inhibitory effect, Wg refines its own expression and also promotes expression of Delta and Serrate to create a positive-feedback loop that maintains Notch signaling and restricts Wg expression to the D/V boundary [18-20]. Wg signaling leads to the expression of proneural genes *achaete* (*ac*) and *scute* (*sc*) specifically within the anterior compartment of the cells flanking the D/V boundary [21], which results in down regulation of Stg and delay of these cells in G2 [9]. The activity of Notch and Wg signaling pathways spatially regulate the activity of bantam micro-RNA and the expression levels of the dMyc to mediate regulation of the E2F transcription factor and the transition of G1 to S-phase [10,11]. Thus the interplay between Wg and N is essential for orchestrating the cell cycle exit across the presumptive margin, which is required for sensory neuron differentiation and development of the wing margin.

Microarray analysis has revealed that the ecdysone signal is associated with modulation of cell cycle regulatory pathways such as Wg, Notch and Dpp during mid-gut morphogenesis [22]. Ecdysone/EcR is also critical for coupling growth and proliferation in the abdominal histoblasts [23]. During larval stages histoblasts grow in a G2 arrested state prior to entering a proliferative stage during pupal metamorphosis [24,25] and the transition to a proliferative state is initiated by ecdysone-dependent activation of the essential G2-M phosphatase String/Cdc25 (Stg) [26]. However, as the *stg* promoter lacks an *EcRE*, further experiments are required to identify the factors mediating the observed transcriptional activation of *stg* required for G2-M progression. Ecdysone can also control animal growth rate via EcR-dependent expression of the growth and S-phase regulator dMyc in the fat body [27]. In this system, loss of EcR function in fat body results in elevated dMyc expression and increased growth, suggesting that EcR signaling normally represses dMyc. However, like the *stg* promoter, *dmyc* lacks an *EcRE* suggesting that the repression of dMyc is unlikely to occur via direct transcriptional regulation by EcR.

Ecdysone pulses therefore control developmental timing and growth of a range of larval tissues [28-30], but how does ecdysone achieve these changes in cell growth and cell cycle progression? In particular, how does ecdysone connect with the major developmental signaling pathways that regulate cell cycle patterning in *Drosophila* imaginal tissues? Here we demonstrate that the release of ecdysone at the end of the third instar is likely to control the timing of the cell cycle delay and initiation of differentiation across the presumptive wing margin via EcR, which is essential for refining *wg* expression and for patterning the cell cycle delay. While dMyc and Stg are key targets of the Notch and Wg pathways, we show that CycB is the major cell cycle target downstream of EcR and Wg. EcR is essential for ensuring CycB expression is maintained along the presumptive wing margin, but not away from the margin and loss of CycB expression in the EcR knockdown clones is dependent on the presence of Wg at the margin.

The *wg* promoter lacks an *EcRE*, suggesting *wg* transcription is unlikely to be directly regulated by EcR. Rather we provide evidence that the effect of EcR on Wg and, therefore, CycB is mediated by the ecdysone/EcR-responsive zinc finger transcription factor Crol. Expression of *crol* is sufficient to restore *wg* repression in the EcR loss of function background, and ChIP revealed that Crol is normally enriched at consensus zinc-finger binding sites within the *wg* promoter. We have therefore added another arm to the mechanism patterning the cell cycle delay across the presumptive wing margin at the end of third instar, whereby parallel pathways can act on Wg to drive a G2 delay; signaling through EcR/Crol-Wg down regulates CycB while interaction between Notch and Wg inhibits Stg. Thus we propose that the pulse of ecdysone at the end of third instar normally ensures proper timing of the cell cycle delay across the presumptive wing margin via EcR and the Wg-repressor Crol, which ensures expression of *wg* is confined to the D/V boundary and controls timing of the G2 delay via CycB.

## Results

### EcR is essential for cell cycle patterning throughout the wing margin

**Figure 2:**
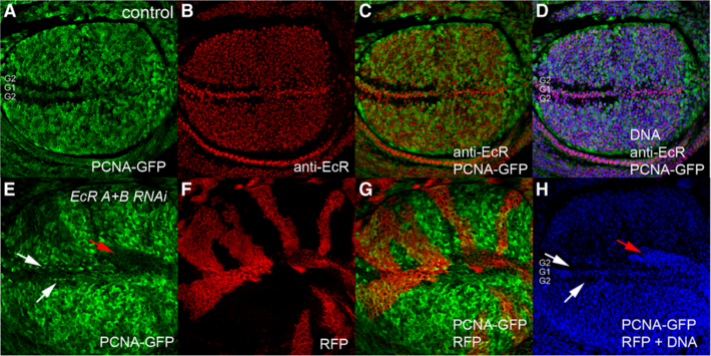
**EcR is required for normal patterning of E2F1 activity in the wing margin. (A**-**D)** - *PCNA-GFP*/+ wing imaginal discs stained with the EcR antibody. PCNA-GFP **(A)** is normally detected in cycling cells of the wing pouch and in the G1 band within the anterior and posterior of the wing margin. EcR staining in red **(B)** overlaps with PCNA-GFP throughout the pouch and the G1 cells of the margin **(C** and **D**, DNA in blue**)**. **(E**-**H)** - *EcR* RNAi flip out clones generated in the *PCNA-GFP* background are marked with CD8-RFP. White arrows mark ectopic G1 cells in the G2 region. The DNA stain in H is for the equivalent confocal section and white arrows show the position of ectopic PCNA-GFP corresponding to clones in the anterior, while the red arrow corresponds to the clone with reduced PCNA-GFP in the posterior.

As EcR is abundantly expressed along the wing margin (Figure 2A-D), we hypothesised that the rise of ecdysone levels at the end of the third instar larval period might be required to pattern cell cycles during this critical stage of wing metamorphosis. In wing imaginal discs, DNA synthesis is coupled with cell division; cells grow in G1, initiate DNA replication and enter S-phase, which is separated from mitosis by G2 phase. To first monitor S-phase progression, we used the *PCNA-GFP* reporter, which gives a read out of E2F1 transcription factor activity and, therefore, indicates whether cells are in late G1 or S-phase [31]. The pattern of E2F1 activity across the apical surface of the wing disc epithelium in a wild type background together with the overlapping pattern of EcR protein is shown in Figure 2 (A-D). PCNA-GFP is normally detected in cycling cells of the wing pouch and in the G1 cells within the anterior and posterior of the wing margin, but decreased in the G2 cells of the anterior margin. EcR protein, detected using the EcR common antibody to both EcR A and B isoforms [32], is ubiquitously expressed throughout the wing pouch, but shows relatively higher levels of expression across the D/V boundary. Thus EcR staining overlaps with PCNA-GFP throughout the pouch and the G1 cells of the margin.

To test whether EcR knockdown affected E2F activity we generated flip out clones [33] using the previously characterised *UAS-EcR* RNAi targeted to both EcRA and EcRB receptors [34]. Across the presumptive wing margin, *EcR* RNAi disrupted E2F1 patterning, in both the anterior cells flanking the boundary, with GFP detected in many of the cells that should normally be delayed in G2 (Figure 2E, white arrows) and also appeared to decrease PCNA-GFP in the posterior margin (Figure 2E, red arrows). The EcR pathway is, therefore, required for normal patterning of E2F1 transcription factor activity across the margin of the presumptive wing blade.

### EcR is required for Wg repression, but only partially regulates E2F1 activity via Wg

**Figure 3:**
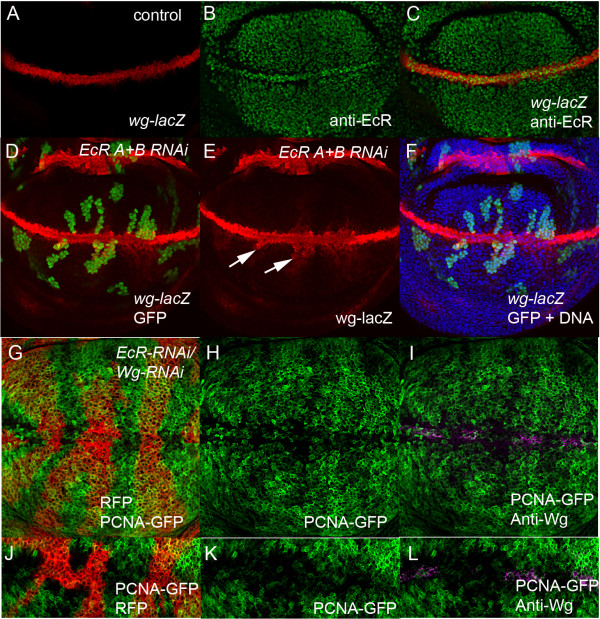
**EcR pathway is required for Wg repression. (A**-**C)** - EcR antibody staining (green) in the *wg-lacZ* background, detected with β-gal (red). **(D**-**F)** - *wg-lacZ* activity (red) in *EcR* RNAi clones marked with GFP. **(G)** - Control pattern of Wg antibody staining (red). **(G**-**I)** - shows *wg* RNAi and *EcR* RNAi double knockdown (marked with CD8-RFP) with PCNA-GFP patterning across the margin, while **(J**-**L)** shows a second example of *wg* RNAi and *EcR* RNAi co-knockdown focusing on the wing margin.

Our previous work revealed a potential link between ecdysone signaling and the Wg pathway, as we demonstrated that the ecdysone-responsive transcription factor Crol [35] is required for repression of Wg in the third instar wing disc [36]. Given that inhibition of the Wg pathway across the margin has been associated with ectopic activation of cell cycle regulators *dmyc* and *stg*, which leads to ectopic cells in S-phase and mitosis [9,10,13,14], we set out to determine whether the disruption to E2F1 activity in *EcR* loss-of-function clones was mediated by Wg. EcR protein is abundant in the *wg* expressing cells (marked by *wg-lacZ)* at the margin, but is also expressed in surrounding non-*wg* expressing cells throughout the wing imaginal disc (Figure 3A-C). Consistent with our previous study using the EcR dominant negative transgenes [36], *EcR* RNAi results in an expansion of *wg* promoter activity (Figure 3D-F, compare with the control Figure 3A) away from the D/V boundary, which further suggests that the EcR pathway is normally required to restrict *wg* expression to the D/V boundary. As previous reports had demonstrated that inhibition of the Wg pathway using TCF-dominant negative (DN) transgenes results in increased S-phase across the D/V boundary [9,10], we tested whether the effect of EcR on E2F1 activity might be mediated by Wg. However, E2F1 patterning across the D/V boundary was not rescued by Wg knockdown in *EcR* RNAi clones (Figure 3G-K), compared with control 2A and EcR knockdown alone in 2E). To ascertain how loss of EcR might lead to disruption of E2F1 activity, we set out to determine whether EcR knockdown impacted other Wg cell cycle targets, including dMyc and Stg [9-11,13,14].

### EcR is not required for dMyc expression but is required for Stg repression across the margin

dMyc is a key mediator of growth and S-phase progression in the wing imaginal disc [10] and previous work has shown that inhibition of Wg pathway activity is sufficient to ectopically activate *dmyc* expression and S-phase throughout the wing margin [10,11]. We therefore tested whether loss of EcR might lead to ectopic E2F1 activity via effects on dMyc abundance using the dMyc antibody (from P. Bellosta and D. Grifoni [37]) on *EcR* RNAi clones (Additional file 1: Figure S1A-D). Although EcR knockdown across the margin results in ectopic E2F1 activity in the G2 band, levels of dMyc are not obviously altered in EcR RNAi clones spanning the G2 region, which suggests the expansion of the Wg domain in the *EcR* RNAi clones is unlikely to alter S phase progression via dMyc.

As Wg signaling also leads to down regulation of the essential mitotic regulator Cdc25 phosphatase String (Stg) across the G2 band of the margin at the level of transcription, we used a *stg-lacZ* enhancer trap to monitor *stg* promoter activity. Distribution of the *stg-lacZ* enhancer trap and Wg protein shows *stg* promoter activity overlapping with Wg in the G1 cells of the margin, decreased in the G2 delayed cells, and abundant throughout the remainder of the pouch (Additional file 1: Figure S2A-D). Surprisingly, rather than leading to decreased *stg* promoter activity, as would be predicted given the expansion of the Wg domain in the *EcR* RNAi clones (Figure 3), EcR knockdown increases *stg-lacZ* activity in clones spanning the margin (Additional file 1: Figure S2E-H). Together the data suggests that disruption to cell cycle patterning across the margin in the *EcR* RNAi clones is unlikely to be due to direct effects on dMyc, E2F or Stg.

### EcR is essential for CycB patterning across the wing margin

**Figure 4:**
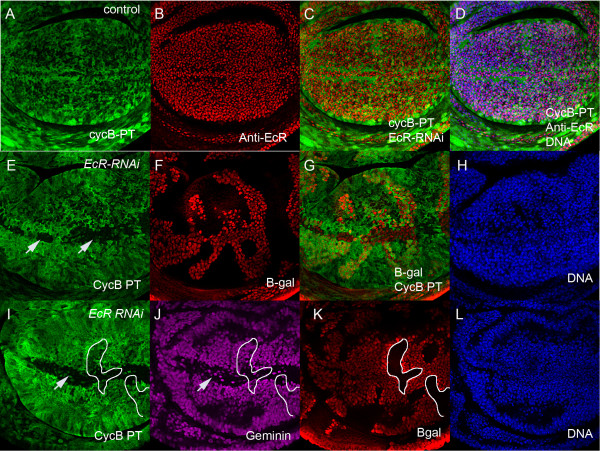
**EcR is essential for CycB expression at the wing margin. (A**-**D)** - Pattern of overlap between the *Cyclin B-GFP* protein trap (*CycB-PT*) in green **(A)** and anti-EcR antibody in red **(B)**. The merge is shown in **(C)** and overlap with DNA in **(D)**. **(E**-**H)** - *EcR* RNAi clones marked with β-gal (red) with *CycB-PT* (green). (**E**) - disruption to the Cyc B pattern across the margin marked with white arrows corresponds to the EcR RNAi clones shown with β-gal in F. The merge is shown in **G** and the presence of cells across the margin lacking CycB shown with DNA in **H**. **(I**-**L)** - Disruption to both *CycB-PT* activity in green **(I)** and the G2-marker Geminin in purple (**J**) for large *EcR* RNAi clones detected with β-gal in red **(K)**. In **(I**-**K)** - the position of two sets of non-EcR knockdown cells is show across the margin to highlight non-autonomous affects of EcR knockdown. The presence of cells across the margin shown with DNA in **(L)**.

The finding that dMyc is not altered and *stg* is ectopically expressed led us to investigate whether EcR might normally modulate cell cycle in the margin via the key G2-M cyclin, Cyclin B, which is also essential and rate-limiting for G2-M progression [38]. For this we first used a *Cyclin B-GFP* protein trap (*CycB-PT,* Carnegie collection CC01846, [39]) to monitor *CycB* expression in the wing. The *CycB-PT* reflects the pattern of CycB protein distribution in the wing (Figure 4A compare with anti-CycB in Additional file 1: Figure S3B) and the anti-EcR antibody and the *CycB-PT* overlap throughout the wing pouch (Figure 4A-D). The result of EcR knockdown is striking, with *EcR* RNAi clones spanning the margin having dramatically decreased *CycB-PT* activity, particularly within the band of cells normally arrested in G2 (Figure 4E-H). To confirm that *EcR* RNAi also affects the distribution of CycB protein in a similar manner to the GFP-protein trap, we used the CycB antibody (From D. Glover [40]). In line with the *CycB-PT* data, EcR knockdown also results in decreased CycB protein across the margin (Additional file 1: Figure S3D-F).

The decreased CycB together with the elevated PCNA-GFP further suggested that *EcR* RNAi clones spanning the G2 region of the margin were experiencing a G1 delay. To further investigate whether the G2 delay was disrupted in EcR loss of function cells at the margin, we co-stained for the DNA-replication inhibitor Geminin, which like CycB is usually abundant from the end of S-phase, peaks in G2 and is degraded at the anaphase-metaphase transition (Additional file 1: Figure S4G,I; [41]). Indeed, consistent with *EcR* RNAi disrupting the G2 delay, we observe decreased Geminin in the presumptive G2 band, with G2 cells only observed at the position normally occupied by the G1 band (Figure 4I-L, marked by a white arrow). Together the cell cycle analysis for *EcR* RNAi clones suggests that EcR is normally required for expression of CycB (Figure 4), but for repression of Stg throughout this region of the margin (Additional file 1: Figure S2). To ensure that *stg* expression was not upregulated as a consequence of the decreased CycB in the *EcR* RNAi clones we generated *CycB* RNAi clones in the *stg-lacZ* background (Additional file 1: Figure S4A-C). As patterning of the *stg-lacZ* reporter was not affected in *CycB* RNAi clones (compare with Additional file 1: Figure S2A-D), elevated *stg* promoter activity is unlikely to be an indirect consequence of CycB down regulation. Thus EcR is normally required to maintain cells in G2 via its ability to activate expression of CycB, which is likely required for the final rounds of G2-M progression across the wing margin. Moreover, the effect of the EcR pathway on CycB is specific to the margin and also results in non-autonomous affects on CycB in cells adjacent to clones spanning the margin (two sets of non-clonal tissue is outlined in white in Figure 4I-K), which suggests this might be mediated by expression of Wg across the D/V boundary.

### The cell cycle delay in EcR loss of function clones is dependent on Wg and CycB

**Figure 5:**
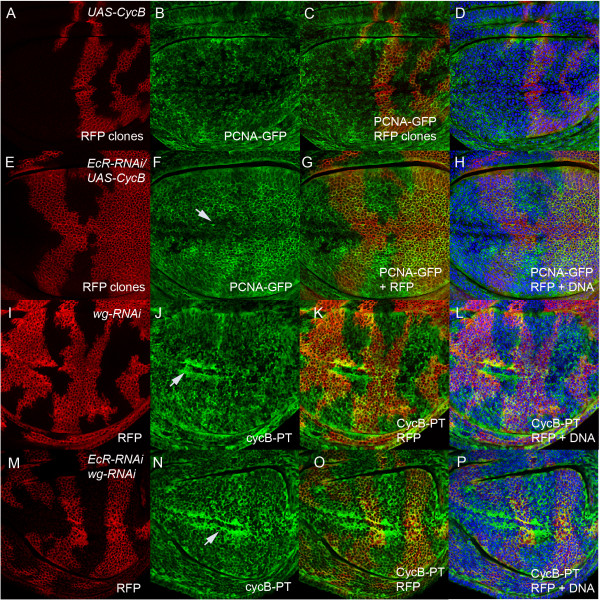
**Overexpression of CycB or knockdown of Wg partially restores cell cycle patterning in *EcR *RNAi clones. (A**-**D)** - *UAS-CycB* clones **(**marked with CD8-RFP in **A)** do not disrupt PCNA-GFP patterning across the anterior margin **(B)**. The merge is shown in **(C)** and overlap with DNA in D. **(E**-**H)** - Overexpression of *CycB* partially restores PCNA-GFP patterning **(F**, see arrow for remaining G1 cells**)** in *EcR* RNAi clones marked with CD8-RFP in **(E)**. The merge is shown in **(G)** and overlap with DNA in **(H)**. **(I**-**L)**- *wg* RNAi clones marked with CD8-RFP **(I)** disrupt *CycB-PT* patterning **(J**, see arrow**)**. The merge is shown in **(K)** and overlap with DNA in **(L)**. **(M**-**P)** - The double *wg* RNAi and *EcR* RNAi clones marked with CD8-RFP **(M)** result in restoration of *CycB-PT* patterning **(N)**. The merge is shown in **(O)** and overlap with DNA in **(P)**.

As S-phase and mitosis are coupled in the wing [42], we speculated that the disruption to E2F1 activity in the *EcR* RNAi clones might be an indirect consequence of the reduced abundance of CycB, and therefore tested whether overexpression of CycB could restore E2F1 activity in the *EcR* RNAi clones (Figure 5). Overexpression of *CycB* in *EcR* RNAi clones partially restored PCNA-GFP patterning across the margin, with PCNA-GFP staining no longer decreased in the clones flanking the posterior margin and only occasional ectopic PCNA-GFP cells in the G2 band of the anterior (Figure 5F, marked by an arrow). Together, this data is consistent with CycB being a key downstream target of EcR for normal patterning of cell cycle across the wing margin.

As *EcR* knockdown is associated with an expansion of the Wg domain and the disruption to *CycB* expression in *EcR* RNAi clones only occurs around the D/V boundary where Wg is abundant, we tested whether we could restore *CycB* expression via co-knockdown of Wg. First, consistent with Wg normally being required for regulating *CycB* expression, the patterning of the *CycB-PT* was disrupted in *wg* RNAi clones spanning the margin (Figure 5I-L). Within the *wg* RNAi clones we observed unpatterned expression broadly across the margin, with ectopic CycB observed in the G1 band. In addition, we observed increased CycB adjacent to the large *wg* RNAi clone across the margin, consistent with Wg being required non-autonomously for repression of CycB (Figure 5J, white arrow). Strikingly, EcR knockdown only results in decreased CycB expression in the presence of Wg, since Wg co-knockdown restores *CycB-PT* activity throughout *EcR* RNAi clones (Figure 5M-P, compare with Figure 4E,I). Therefore, in the absence of EcR, Wg is elevated in the G2 band and CycB expression is lost. However, in the absence of Wg, down regulation of CycB no longer occurs in the EcR knockdown cells, which suggests Wg is required for EcR-dependent patterning of CycB at the margin. Thus we have identified a novel pathway for regulating cell cycle patterning across the margin, whereby EcR normally activates CycB in the G2 band by modulating the abundance of Wg.

### Crol restores *wg* repression in EcR loss of function cells

**Figure 6:**
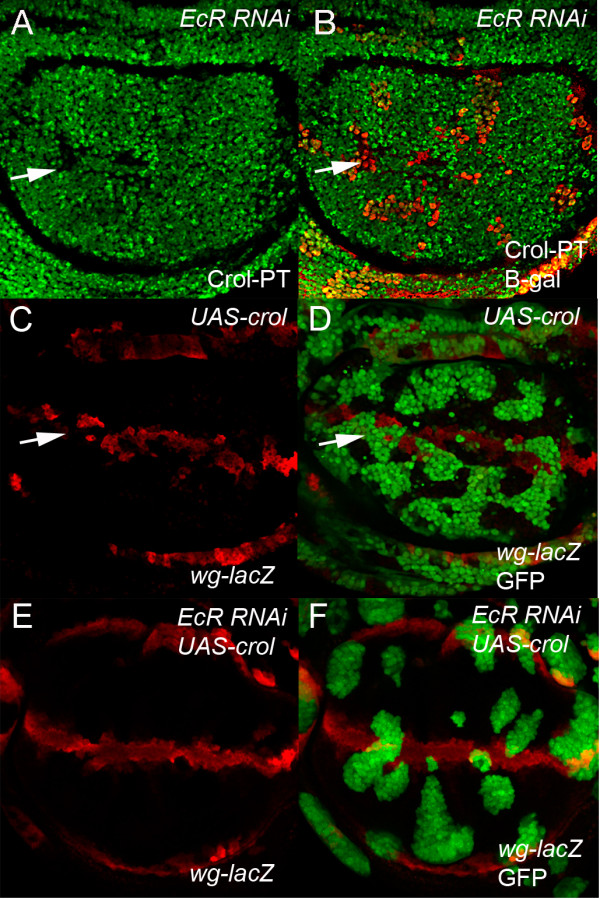
**Crol restores repression of *wg *expression in EcR knockdown. (A**-**B)** - *Crol-GFP* protein trap activity **(A)** is reduced in *EcR* RNAi clones marked with β-gal in red for the merge **(B)**. **(C**-**D)** - *wg-lacZ* activity marked with β-gal in red **(C)** is reduced in *UAS-crol* clones marked with GFP in the merge **(D)**. **(E**-**F)** - *UAS-crol* restores repression of *wg-lacZ* activity in the *EcR* RNAi clones.

The absence of EcR response elements in the *wg* promoter suggests that *wg* is unlikely to be a direct transcriptional target of EcR. We have previously shown that the ecdysone inducible-zinc finger transcription factor Crooked Legs (Crol) drives proliferation in *Drosophila* via effects on *wg* expression [36]. Like EcR, *Crol* is normally expressed throughout the wing imaginal disc with reduced levels in the G2 cells of the margin [36]. Crol is responsive to the ecdysone pulse [35] and consistent with the multiple *EcR* binding sites in the *crol* promoter (data not shown), EcR is necessary for normal levels of *crol* expression (Figure 6A-B). In line with Crol being sufficient for *wg* repression, overexpression of *crol* leads to disruption of the *wg* expression domain and decreased *wg-lacZ* activity (Figure 6C-D). Although the *wg-lacZ* band was still disrupted when Crol is overexpressed in the EcR loss of function background, expansion of *wg-lacZ* expression was no longer detected in clones away from the margin (Figure 6E-F). Thus overexpression of *crol* can partially restore *wg-lacZ* activity to the D/V boundary in the EcR loss-of-function clones, which suggests EcR normally regulates *wg* expression via Crol across the wing margin.

### Crol may mediate the effect of EcR on *wg* transcription

**Figure 7:**
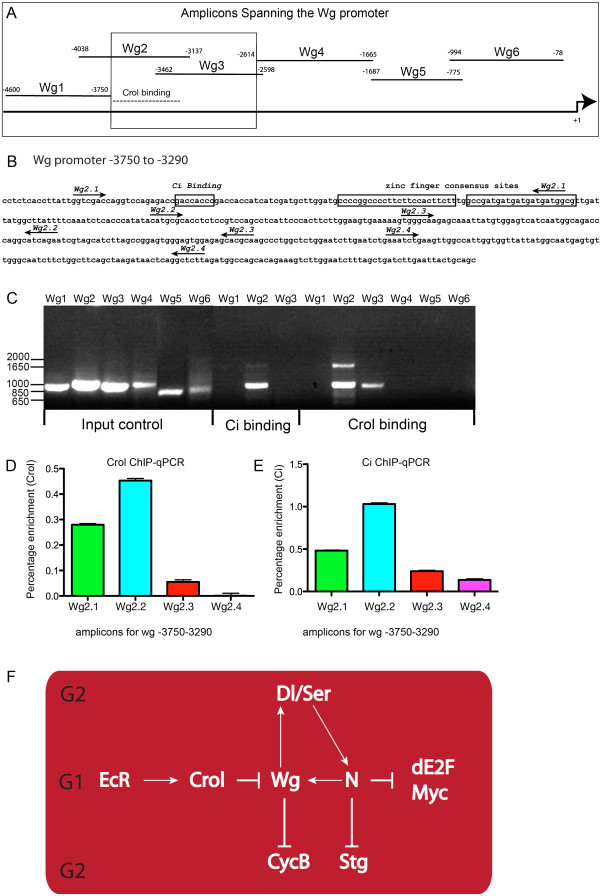
**Crol is enriched across consensus zinc finger binding sites in the *wg *promoter. (A)** - 6 overlapping primer sets spanning 5 kb of the *wg* promoter, *wg* transcription start site +1. **(B)** - region of *wg* promoter containing overlapping amplicons for *wg* (Wg primers 1–6) used for ChIP with anti-Crol or Ci, the boxed region highlights amplicons positive for Crol enrichment **(**see gel in **C)** and the dashed grey line shows the region of the *wg* promoter containing the zinc finger and Ci consensus sites shown in 7B. **(B)** - Position of primers for ChIP-qPCR (Wg2.1-Wg2.4) are marked with arrows. The *in vitro* defined Ci binding site and consensus zinc finger binding sites are boxed and labelled. **(C)** – DNA gel electrophoresis of ChIP-PCR across the Wg promoter using Wg primers (as marked in 7) for Input DNA; Ci ChIP or Crol ChIP as marked. **(D**-**E)** Fine mapping of Crol and Ci enrichment using ChIP-qPCR for small amplicons - Wg2.1-2.4 shown in **(B)**. **(D)** - ChIP for Crol - percentage enrichment and error for percentage enrichment for Crol are as follows; Wg 2.1 (0.28 ± 0.0045); Wg 2.2 (0.453 ± 0.0083); Wg 2.3 (0.055 ± 0.0082) and Wg 2.4 (0.00116 ± 0.0095). **(E)** - ChIP for Ci - percentage enrichment and Error for percentage enrichment for Ci are as follows; Wg 2.1 (0.482 ± 0.0066); Wg 2.2 (1.031 ± 0.0141); Wg 2.3 (0.24 ± 0.0103) and Wg 2.4 (0.137 ± 0.019). **(F)** - Model for regulation of cell cycle patterning across the anterior wing margin by EcR.

The data above suggests the zinc finger transcription factor Crol might provide a link between the ecdysone pulse and repression of *wg* transcription across the wing margin. We have previously shown that Crol is unlikely to affect *wg* transcription indirectly via effects on the Notch or Hh pathways [36] and, therefore, set out to test whether Crol might directly inhibit *wg* transcription in larval imaginal tissue by conducting ChIP with overlapping primer sets spanning the < 5kb *wg* promoter (Figure 7). As reporter constructs corresponding to the region covered by the first 3 primer sets (−4600 to −2614) had been previously shown to be bound by Ci in *Drosophila* S2 cells *in vitro*[43-45], we carried out ChIP with these primer sets using the Ci antibody as a positive control. We detected enrichment for Ci using primer set Wg2, but not on primer set Wg1 or Wg3, which narrows down the binding to between −3750 and −3462 of the *wg* promoter (Figure 7A, C). ChIP carried out for Crol, revealed enrichment for overlapping primer sets Wg2 and Wg3 (Figure 7A, C), suggesting Crol binds the *wg* promoter between −3750 and −2614. Analysis of the 1160 bp sequence contained within this portion of the *wg* promoter using the Zinc Finger Tools (ZF Tools) website (http://www.zincfingertools.org) to identify contiguous sites with a minimum target size of 24 bp (for 8 finger binding) [46] revealed 2 strong consensus zinc finger binding sites within this region of the *wg* promoter (−3682, -3656 boxed in Figure 7B).

To determine whether Crol or Ci might normally bind these consensus zinc-finger binding sites we designed overlapping primer sets for ChIP-qPCR for this 1160 bp region i.e. between −3750 and −2614 (Wg2.1-Wg2.4 shown in Figure 7B). Significant enrichment was observed for both Crol and Ci across the 2 most 5′ amplicons (Wg2.1 and Wg2.2 primers) in the region containing 2 zinc finger consensus sites, compared with the more 3′ amplicons (Figure 7D,E). Thus the *wg*-repressor Crol and the *wg*-activator Ci are enriched within overlapping regions of the *wg*-promoter and it will be of future interest to determine whether the fine-tuning of *wg* expression involves interaction between Ci and Crol. Together this data suggests that EcR normally maintains cell cycle across the wing margin by activating expression of the Crol transcription factor, which in turn binds the *wg* promoter to repress transcription (Figure 7F). Thus we have identified a critical function for the ecdysone pathway in refining expression of the Wg morphogen and, as a consequence, ensuring proper timing of cell cycle exit at the margin via expression of the key mitotic regulator CycB.

## Discussion

We have identified the essential mitotic cyclin, CycB as the key target of the ecdysone/EcR system that normally ensures cells remain competent to complete their final divisions during the early pupal stage by activating expression of the Crol transcription factor (Figure 7F). Early studies demonstrated that Crol is activated in late larval imaginal discs by the steroid hormone ecdysone [35]. More recently Crol was identified in a genetic screen for factors capable of disrupting the establishment and/or maintenance of heterochromatic silencing in either cycling and differentiated cells [47], consistent with a transcriptional role for Crol in maintaining active chromatin states of many genes specific to cycling cells. Although this screen identified many chromatin and RNA processing factors capable of de-repressing silencing, most factors behaved as de-repressors in both cycling and differentiated cells. Interestingly, only two factors, Crol and the bantam micro RNA, were found to de-repress silencing in cycling, but not differentiated cells, both of which have now been implicated in regulating cell cycle across the wing margin (this work, [11,36,48]).

The rise of ecdysone levels at the end of the third larval instar [1] coincides with reduced rates of cell division across the wing disc [5,6] and increased expression of proneural genes in clusters of cells, which then form the sensory organ precursor (SOP) lineage required for bristles development along the wing margin [7,8]. At the beginning of the pupal period, the bristle precursors complete their final cell divisions and by 24 hours APF all epithelial cells have arrested in G1 [5,6]. Our data suggests that loss of EcR across the margin results in a premature arrest of these cells in G1. EcR is therefore required for coordinating the delay in cell cycle progression with differentiation of the sensory neurons during wing metamorphosis. Indeed previous studies have shown that EcR is required for repressing the differentiation of the SOPs in the wing margin, as RNAi results in ectopic senseless expression [34]. The finding that sensory neurons in the margin differentiate precociously is consistent with our findings that suggest EcR coordinates the onset of sensory neuron development by maintaining cell cycle gene expression across the margin. Furthermore, in the studies by Schubiger et al. (2005), loss-of-function EcR only resulted in precocious differentiation of SOPs not ectopic differentiation, which suggests that this is mediated by a second factor normally expressed across the margin, and our work suggest that this is likely to be Wg. Consistent with this idea, ectopic activation of the Wg pathway also results in ectopic SOPs [21]. The data here demonstrates Wg is a critical target in mediating the effect of the EcR signaling pathway on cell cycle patterning in the wing imaginal disc. Inhibition of the EcR pathway results in ectopic *wg* transcription and expansion of the band of Wg protein comprising the dorsal-ventral boundary of the wing imaginal disc. Thus EcR is normally required for constraining the expression of Wg to the D/V boundary of the wing imaginal disc in order to pattern the final rounds of cell division required for differentiation and development of the wing margin.

Previous work has demonstrated that the G2 cell cycle delay across the wing imaginal disc is mediated by down regulation of Stg in response to activity of the Wg and Notch pathways [9,11]. At the D/V boundary, Notch can activate *wg* expression [15,17] and Wg signaling activates *ac/sc* to lead to down regulation of *stg* and delay of the cells flanking the boundary in G2 [9]. Thus we originally predicted that *EcR* RNAi clones, which show an expansion of Wg away from the margin, might display down regulation of Stg. Conversely, we observed ectopic Stg in the G2 band of the margin, which raises the question of how expansion of the Wg domain in the EcR knockdown result in ectopic *stg*? This could be due to the ability of Wg to repress N away from the D/V boundary [11,48], whereby the expansion of Wg in the EcR knockdown might result in ectopic Notch activity, which would lead to repression of *ac/sc* expression and up regulation of *stg* in the non-boundary G2 band [11]. In the EcR RNAi clones, Notch would still be abundant at the D/V boundary to autonomously delay cells in G1, via down regulation of dMyc and Bantam, however, in the absence of EcR these cells would be unable to progress through G2-M due to the decreased expression of CycB (Figures 2 and 4). Furthermore previous work has shown that *stg* overexpression only triggers cell-cycle progression in embryonic and imaginal cells previously arrested in G2 [5,49,50], but not in G1-arrested cells [51]. Thus the cells spanning the margin in the EcR knockdown are predominantly delayed in G1. The interplay between EcR/Wg and N/Wg is therefore likely to be essential for orchestrating the cell cycle exit across the presumptive margin, which is required for sensory neuron differentiation and development of the wing margin.

Ecdysone-dependent control mechanisms have been reported for restricting growth to the juvenile period, where ecdysone controls growth rate via dMyc in the fat body [27]. In contrast to the role of EcR as a CycB activator in the wing margin, EcR signaling normally represses dMyc and its downstream targets in the fat body. Furthermore, the ability of circulating ecdysone to control dMyc expression during the pupal stage was found to be specific to the fat body. For example, *dmyc* mRNA levels were elevated in fat body after reducing the level of circulating ecdysone, via inhibition of PI3K pathway in the prothoracic gland, but at this stage global changes to *dmyc* levels were not detected in wing imaginal discs [27]. Consistent with this, we did not observe changes in dMyc levels in *EcR* RNAi clones throughout the third instar wing (Additional file 1: Figure S1B-C). Thus the effect of ecdysone on this key growth regulator appears to be tissue specific; resulting in down regulation of *dmyc* expression in the fat body, but not in the wing disc either during the larval or pupal stage.

The ecdysone pulse at the larval-pupal transition is required for the *stg* transcription triggering histoblast proliferation at the onset of abdomen metamorphosis [26]. In contrast to the wing epithelium, during larval stages histoblasts grow in a G2 arrested state prior to entering a proliferative stage during pupal metamorphosis [25,52]. During larval stages, the arrested histoblasts accumulate cellular mass and the transition to a proliferative state is initiated by ecdysone-dependent *stg* transcription [26]. The latter can occur because the larval histoblasts have preaccumulated stores of the G1 cyclin, Cyclin E, which is sufficient to trigger S-phase after mitosis. In the histoblast, overexpression of Stg, but not Cyclin A, Cyclin B, or Cdk1, can trigger their premature hyper proliferation in larval stages [26]. Although ecdysone is necessary to trigger histoblast proliferation [23] up regulation of *stg* transcription in larval stages bypasses the requirement for ecdysone pathway activity. Thus ecdysone is important for coupling growth and proliferation in abdominal histoblasts [26].

It will be of interest to determine whether the changes reported in *stg or dmyc* are due to direct transcriptional effects of EcR or are mediated by changes to developmental signaling. Our findings in the imaginal tissues suggest that EcR might regulate cell cycle genes in larval histoblasts and fat body indirectly, by modulating upstream developmental signaling pathways. As noted by Delanoue (2010), the lack of consensus binding sites for EcR/Usp (EcREs) in the *dmyc* promoter region suggests that *dmyc* is not a direct target of EcR-mediated gene repression in the fat body, but rather that EcR signaling indirectly controls *dmyc* transcription. Although the fat-specific target of EcR leading to altered *dmyc* expression is unknown, here we have shown that in the wing imaginal disc EcR can activate the Wg-repressor Crol, which is required for repression of the Wg morphogen.

## Conclusions

Despite evolutionary divergence, functional studies suggest that the estrogen steroid hormone pathway is functionally similar to the ecdysone pathway [53-55]. Aberrant estrogen signaling is associated with a variety of hormone-dependent diseases, including cancer [56]. Moreover, in human colon cancer, estrogen receptor (ER) signaling is documented as an inhibitor of the Wnt pathway [57], which has long been implicated in initiation and progression of colorectal cancer [58], however, the mechanism by which estrogen modulates Wnt signaling is currently unknown. Our discovery that EcR represses the Wg pathway via an intermediate zinc-finger transcription factor may, therefore, have broader implications for studies of Wnt pathway regulation by estrogen/ER in colorectal cancer.

## Methods

### Drosophila strains

The following fly stocks were obtained from the Bloomington Stock Centre; *UAS-CycB*; *wg-lacZ* (*P{en1}wg*^*en11*^; [59]); *UAS-EcR* RNAi [34]. *wg-RNAi* (v13351; v13352) was obtained from the Vienna *Drosophila* RNAi Centre, http://www.vdrc.at/[60]. The *CycB* (CC01846) and Crol (CB 03039) GFP protein trap lines were from The Carnegie Fly Trap collection [39]. All other lines were as follows; *Act < CD2 < GAL4 UAS-GFP* (L. Johnston), *PCNA-GFP* (R. Duronio*)* and *stg-lacZ* (*P(2) Stg 01235*) (Sveged, S11525), *UAS-crol* transgenic lines (Mitchell et al., 2008).

### Immunohistochemistry and microscopy

For all flip-out clones larvae were heatshocked for 20 minutes at 37°C 48 hours after egg deposition. Larvae were raised at 25°C for 72 hours to allow development to the third larval instars prior to dissection. All other larvae were dissected 120 hours after egg deposition. Antibody staining was carried out as described previously [36,61]. Antibodies used were: Cyclin B (D. Glover; [40]), anti-Geminin [41], anti-β gal (Sigma), anti-GFP (to detect PCNA-GFP only, Invitrogen), Anti-Wingless 4D4 (DSHB), EcR common (DSHB). Image preparation and analysis are conducted in Adobe Photoshop CS2 Version 9.0 ©, ImageJ.

### ChIP across *wg* promoter

ChIP was carried out using the ChIP Assay Kit (Upstate Biotech) as described previously [61]. For each ChIP sample (for PCR or qPCR), 200 larval heads were fixed in 4% paraformaldehyde in PBS for 40 minutes. ChIP followed by PCR was first carried across the entire *wg* promoter after sonication for genomic fragments between 400bp-1kb (not shown). IP was carried out using either Ci or the Crol antibody, which we have shown to specifically detect Crol protein [36]. The primers used for PCR to test binding of Crol and Ci across the 4625bp span of the *wg* promoter were; Primer set 1 (827bp product −4600 to −3750) 5′ agcgtggacgatgataatgc 3′ and 5′ tcgaccaataaggtgagagg 3′; Primer set 2 (901 bp product −4038 to −3137) 5′ aagtgcgtgaaccatgtcg 3′ and 5′ tggcagatcaacaccattagg 3′; Primer set 3 (864bp product −3462 to −2598) 5′ ggccattggtggttattatgg 3′ and 5′ tgtcctgtcagcagaattgg 3′; Primer set 4 (949bp product −2614 to −1665) 5′ ttctgctgacaggacacagc 3′ and 5′ atggcagatagcgagagtgg 3′; Primer set 5 (726bp product −1687 to −775) 5′ ccaccactctcgctatctgc 3′ to 5′ ttcctcatccattgcttgg 3′; Primer set 6 (826bp product −994 to −78) 5′ gccaagcaatggatgagg 3′ to 5′ ggtcttcgtcttcggatcg 3′.

For ChIP-qPCR 200 heads were prepared as above, but sonication was performed to generate 100 bp genomic fragments (not shown). Quantitative real-time PCR (q-PCR) was carried out in triplicate using the SYBR Green PCR Master Mix (Applied Bio systems) and the 7900HT Fast Real-Time PCR System (Applied Bioscience). The primers for qPCR were; qPCR Primer set wg2.1 - 5′ggtcgaccaggtccagagaccg 3′ and 5′cgccatcatcatcatcatcggccaaa 3′; qPCR Primer set wg2.2 - 5′ catgcgcacctctccgtccag 3′ to 5′ tctgatgcctgggtctgcca 3′; qPCR Primer set wg2.3 - 5′ tgggcaagagcaaattatgtggagtca 3′ to 5′ ccagagccagggcttgcgtg 3′; qPCR Primer set wg2.4 - 5′ aaatctgaagttggccattggtggt 3′ to 5′ tctgtgctggccatctaagagcct 3′ (the position of each primer in the refined region of Crol binding is shown in Figure 7). The data analysis was conducted with Sequence Detection Systems v2.3 (Applied Bio systems). Enrichment was determined by normalising signal to input using the “Percent Input Method” [62], which includes normalization for both background levels and input chromatin. The input sample was purified, non-immuno precipitated, sheared chromatin; target was immuno precipitated sheared chromatin; and background was from an immunoprecipitation with non-specific IgG antibody. To normalise to background, the signal obtained for the non-specific IgG for each primer set was subtracted from the ChIP signal from either the Ci or Crol antibody.

## Competing interests

The authors have no financial or non-financial competing interests.

## Authors’ contributions

NM, JL, OZ, NC, AL and LQ conducted experiments. LQ wrote the manuscript. All authors read and approved the final manuscript.

## Supplementary Material

Additional file 1: Figure S1 dMyc protein is not decreased in EcR knockdown. A - dMyc antibody staining on control*.* B-D - *EcR* RNAi clones with anti-dMyc staining. **Figure S2.** EcR is required to represses *stg* in the margin. A-D - Wg staining in *stg-lacZ* enhancer trap background. E-H - *EcR* RNAi clones in the *stg-lacZ* background. **Figure S3.** EcR overexpression disrupts CycB patterning across the margin. A-C - co-staining of control wing dics with Wg and CycB. D-F - *EcR* RNAi clones stained with CycB antibody. G-H - co-staining for Geminin and Cyclin B across the wing imaginal disc. **Figure S4.** CycB knockdown does not affect stg-lacZ activity across the wing margin. A-C - CycB RNAi clones in the *stg-lacZ* background. Click here for file
