# Supplementary material for: The Ecdysone receptor constrains wingless expression to pattern cell cycle across the Drosophila wing margin in a cyclin B-dependent manner
Source: BMC Dev Biol. 2013 Jul 13;13:28. doi: 10.1186/1471-213X-13-28 (PMC3720226; doi:10.1186/1471-213X-13-28)

Supplemental Figures

Supplemental Figure 1 - dMyc protein is not decreased in EcR knockdown. A - dMyc antibody staining on control. B-D - *EcR* RNAi clones with anti-dMyc staining.

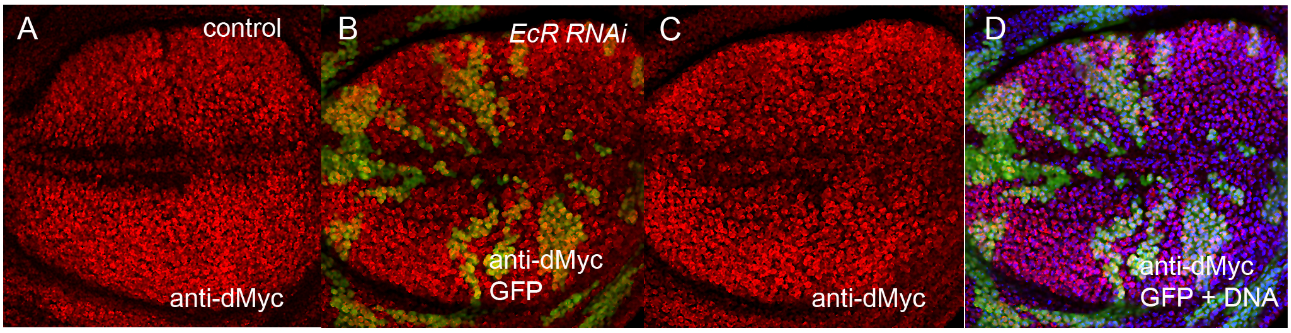

Supplemental Figure 2 - EcR is required to represses *stg* in the margin. A-D - Wg staining in *stg-lacZ* enhancer trap background. E-H - *EcR* RNAi clones in the *stg-lacZ* background.

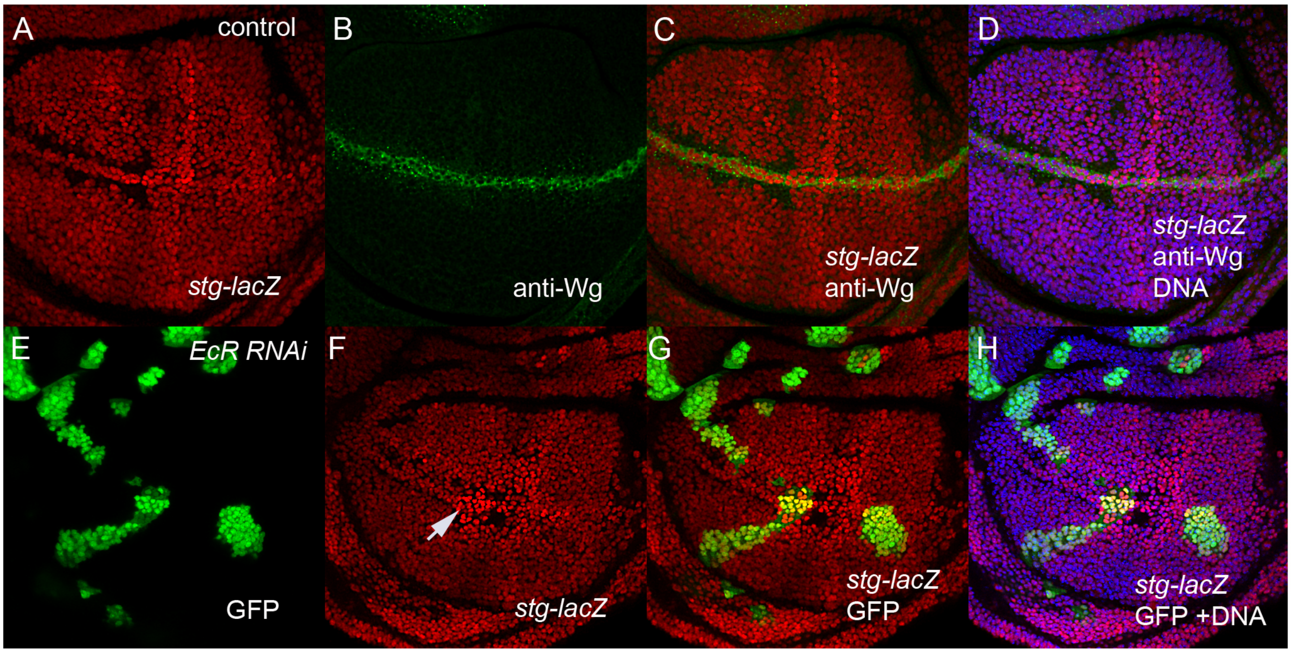

Supplemental Figure 3 - EcR overexpression disrupts CycB patterning across the margin. A-C - co-staining of control wing dics with Wg and CycB. D-F - *EcR* RNAi clones stained with CycB antibody. G-H - co-staining for Geminin and Cyclin B across the wing imaginal disc.

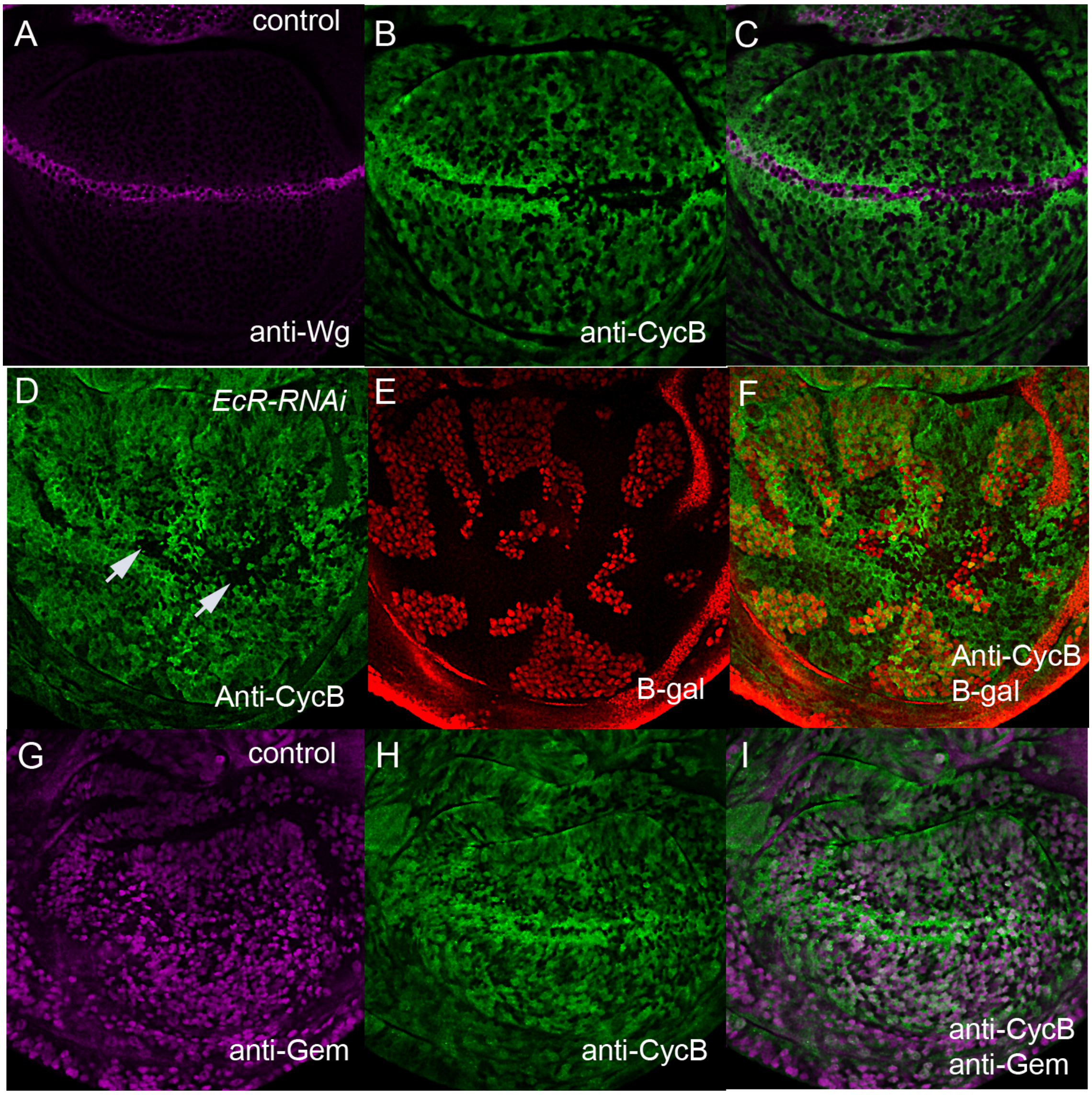

Supplemental Figure 4 - CycB knockdown does not affect stg-lacZ activity across the wing margin. A-C - CycB RNAi clones in the *stg-lacZ* background.

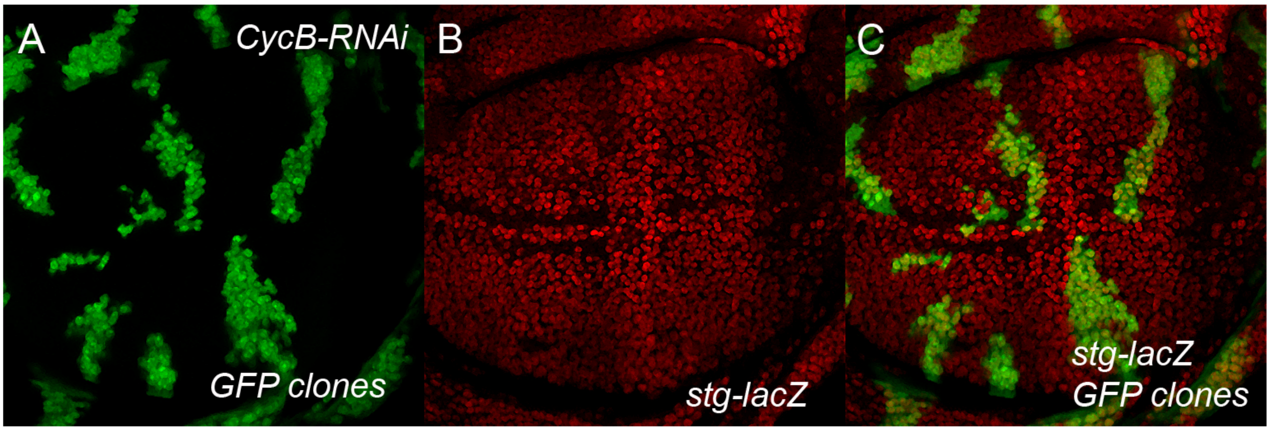

Supplement: Additional file 1: Figure S1 — dMyc protein is not decreased in EcR knockdown. A - dMyc antibody staining on control. B-D - EcR RNAi clones with anti-dMyc staining. Figure S2. EcR is required to represses stg in the margin. A-D - Wg staining in stg-lacZ enhancer trap background. E-H - EcR RNAi clones in the stg-lacZ background. Figure S3. EcR overexpression disrupts CycB patterning across the margin. A-C - co-staining of control wing dics with Wg and CycB. D-F - EcR RNAi clones stained with CycB antibody. G-H - co-staining for Geminin and Cyclin B across the wing imaginal disc. Figure S4. CycB knockdown does not affect stg-lacZ activity across the wing margin. A-C - CycB RNAi clones in the stg-lacZ background. [file 1471-213X-13-28-S1.pdf]
